# An artificial intelligence model to identify snakes from across the world: Opportunities and challenges for global health and herpetology

**DOI:** 10.1371/journal.pntd.0010647

**Published:** 2022-08-15

**Authors:** Isabelle Bolon, Lukáš Picek, Andrew M. Durso, Gabriel Alcoba, François Chappuis, Rafael Ruiz de Castañeda

**Affiliations:** 1 Institute of Global Health, Department of Community Health and Medicine, Faculty of Medicine, University of Geneva, Geneva, Switzerland; 2 Department of Cybernetics, Faculty of Applied Sciences, University of West Bohemia, Pilsen, Czechia; 3 PiVa AI s.r.o, Plzeň, Czechia; 4 Department of Biological Sciences, Florida Gulf Coast University, Fort Myers, Florida, United States of America; 5 Division of Tropical and Humanitarian Medicine, Geneva University Hospitals and University of Geneva, Geneva, Switzerland; 6 Médecins Sans Frontières—Doctors Without Borders, Geneva, Switzerland; 7 Department of Community Health and Medicine, Faculty of Medicine, University of Geneva, Geneva, Switzerland; Universidad de Costa Rica, COSTA RICA

## Abstract

**Background:**

Snakebite envenoming is a neglected tropical disease that kills an estimated 81,000 to 138,000 people and disables another 400,000 globally every year. The World Health Organization aims to halve this burden by 2030. To achieve this ambitious goal, we need to close the data gap in snake ecology and snakebite epidemiology and give healthcare providers up-to-date knowledge and access to better diagnostic tools. An essential first step is to improve the capacity to identify biting snakes taxonomically. The existence of AI-based identification tools for other animals offers an innovative opportunity to apply machine learning to snake identification and snakebite envenoming, a life-threatening situation.

**Methodology:**

We developed an AI model based on Vision Transformer, a recent neural network architecture, and a comprehensive snake photo dataset of 386,006 training photos covering 198 venomous and 574 non-venomous snake species from 188 countries. We gathered photos from online biodiversity platforms (iNaturalist and HerpMapper) and a photo-sharing site (Flickr).

**Principal findings:**

The model macro-averaged F1 score, which reflects the species-wise performance as averaging performance for each species, is 92.2%. The accuracy on a species and genus level is 96.0% and 99.0%, respectively. The average accuracy per country is 94.2%. The model accurately classifies selected venomous and non-venomous lookalike species from Southeast Asia and sub-Saharan Africa.

**Conclusions:**

To our knowledge, this model’s taxonomic and geographic coverage and performance are unprecedented. This model could provide high-speed and low-cost snake identification to support snakebite victims and healthcare providers in low-resource settings, as well as zoologists, conservationists, and nature lovers from across the world.

## Author summary

Snakebite kills 81,000–138,000 people a year and disables another 400,000 mainly in tropical and subtropical Africa, Asia and Latin America. We must be able to correctly identify biting snakes if we want to understand the diversity of snakes that threatens different populations in endemic countries. This is key to improve snakebite epidemiological data and to ensure the appropriate distribution of antivenoms in a given country and the specific treatment of patients with these antivenoms when bitten by prevalent snakes. However, snakes are diverse and healthcare providers lack the expertise to identify them, i.e., victims carry the snake to the hospital or take photos. Here we used thousands of snake photos from across the world and computer vision to develop an AI model to classify snakes. We show for the first time that AI can accurately classify a large diversity of venomous and non-venomous snakes from across the world including lookalike species from snakebite endemic countries. This study sets the foundation for developing global, regional or national snake identification support systems for snakebite epidemiologists and healthcare providers, herpetologists, and the general public.

## Introduction

More than five million snakebites occur globally every year. Venomous snakes cause about half of these bites and kill 81,000–138,000 people and disable another 400,000 in low-resource settings in Africa, Asia, and Latin America [1]. In 2019, the World Health Organization (WHO) launched a roadmap to develop safe, effective, and accessible antivenoms and halve snakebite envenoming (snakebite hereafter) burden by 2030 [1]. Achieving this also depends on improving snakebite diagnosis at the snake species level and better understanding of snake diversity and distribution in snakebite endemic areas [1–3]. Globally, there are over 3,900 snake species [4], about 700 of these are venomous and 292 are medically important venomous species (MIVS) according to WHO [1].

We must correctly identify venomous and non-venomous biting snakes to ensure appropriate distribution of antivenoms and treatment of victims where most needed [2, 3, 5–8]. Misidentification of biting snakes can result in imprecise, unnecessary, and potentially unsafe use of antivenom [9, 10], while unnecessary or incorrect use of antivenoms wastes this scarce and often expensive treatment [8, 11].

Molecular snake identification techniques (e.g., immunoassays that detect venom antigens in snakebite victims) have limitations, and their deployment in low-resource settings remains to be seen [2, 10]. When victims or relatives bring the snake to the health facility or provide a photo, healthcare providers, who are generally not trained in herpetology, often struggle to taxonomically identify the snake [9, 12, 13]. They watch for victims’ symptoms to determine the type of envenomation, infer the associated biting snake, and subsequently decide on the treatment. This syndromic approach has limitations (e.g. syndromic misclassification) and can be complemented by direct snake identification [5, 9, 14].

Computer vision techniques have been developed to identify birds (e.g., Merlin Bird ID app recognizes over 7,500 species) and other animals like fish and butterflies [15], yet few initiatives seek to identify snakes and they are limited to certain taxonomic groups or geographic areas (e.g., [16–18]). In this study, we developed and tested the performance of a computer vision model to classify a large diversity of snakes using thousands of snake photos from across the world publicly available on open biodiversity platforms (iNaturalist and HerpMapper) and another online platform (Flickr)[19, 20]. We further showed the high average per-country accuracy of this algorithm and its capability to distinguish sympatric lookalike species. We also investigated the role of geographical information in fine-tuning the accuracy of snake species identification and if the phenomenon of “unreasonable effectiveness” of noisy data for fine-grained recognition applies to snake recognition [21, 22].

## Methods

### Snake photo datasets

We used a subset of the world’s largest snake photo dataset, described in detail in Durso et al., 2021 [20], which we provided within the snake species identification challenge SnakeCLEF2021. This challenge is part of LifeCLEF21, the Conference and Labs of the Evaluation Forum (CLEF) that proposes data-oriented challenges related to the identification and prediction of biodiversity [23].

The training dataset contains 386,006 photos belonging to 772 snake species from 188 countries and all continents except Antarctica. Most of the photos (87%) come from the online biodiversity platforms iNaturalist (www.inaturalist.org) and HerpMapper (www.herpmapper.org). For species with the fewest images, we further extended the dataset by scraping data from Flickr (13% of the total). Although we attempted to reduce this, Flickr provided us with a heterogeneous and noisy source of data, including imprecise photo labels and photos of non-snake subjects (e.g., captive snakes, photos of snake habitat).

Within the training set, 772 of the world’s 3,921 species (±20%) had at least ten photos, our threshold for inclusion. The dataset has a marked long-tailed class distribution, where the most frequent species (*Thamnophis sirtalis*) is represented by 22,163 photos and the least frequent by just ten.

The 772 species are classified into 18 families (1–418 species per family in the training set) and 269 genera (1–32 species per genus in the training set). They include 198 out of the 292 (68%) medically important venomous snake species (MIVS) according to WHO’s classification [24] and 574 non-MIVS.

For testing, we used the test set from the SnakeCLEF2021 competition with 23,673 photos submitted to the iNaturalist platform within the first four months of 2021, allowing us to compare the performance with other studies (see S1 and S2 Tables for more details on the training and testing datasets and S3 Table and S1 and S2 Figs for MIVS included in the datasets).

Considering that all snake species have distinct, largely stable geographic ranges, with a maximum of 126 species of snakes occurring within the same 50×50km^2^ area [25], geographical information could play a crucial role in correct snake species identification. To evaluate this, we gathered two levels of geographical label (i.e., country and continent) for approximately 87% of the data.

**Fig 1:**
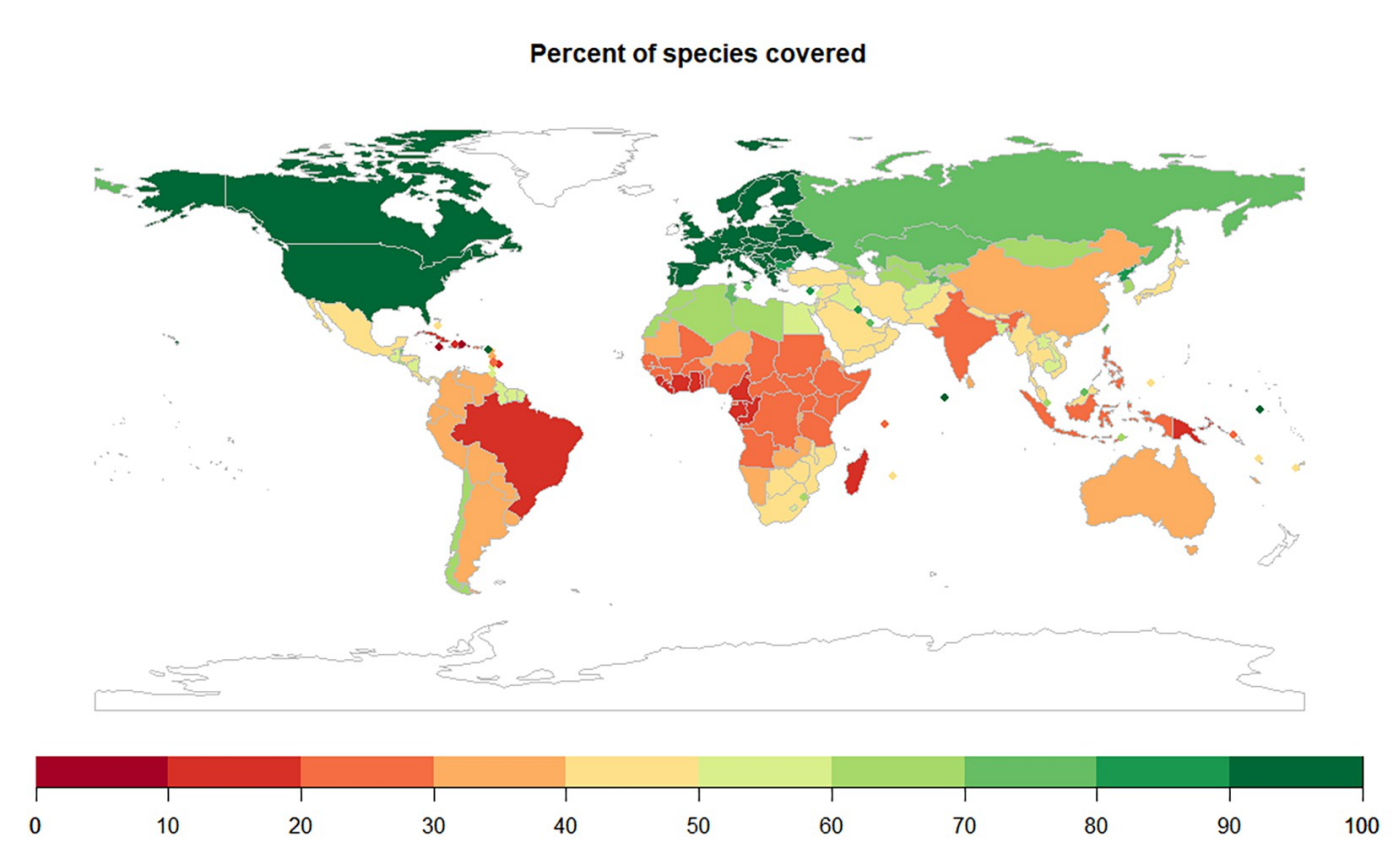
Percent of snake species represented in the training dataset for each country. The map was drawn using R package rworldmap v 1.3.6 [26]. See S1 Data for underlying values.

The vast majority (77%) of photos came from the United States and Canada, with 9% from Latin America and the Caribbean, 5.7% from Europe, 4.5% from Asia, 1.8% from Africa, and 1.5% from Australia/Oceania. Bias at smaller spatial scales undoubtedly exists as well [20], largely due to where participants in citizen science projects and other snake photographers are concentrated. Nevertheless, snake species from nearly every country were represented, with 46/215 (21%) of countries having all of their snake species represented, mostly in Europe. Nearly half of all countries (106/215; 49%) had more than 50% of their snake species represented (Fig 1).

A small proportion of photos (ca. 1–2%), particularly from Flickr, show captive snakes that are kept outside of their native range (e.g., North American *Pantherophis guttatus* in Europe or Australian *Morelia viridis* in the USA). We opted to retain these for three reasons:

1. Users of an automated identification system may wish to use it on captive snakes (e.g., in the case of customs seizures) [27].
2. Bites from captive snakes may occur (although the identity of the snake would normally be clear in this case) (e.g., [28]).
3. Captive snakes sometimes escape and can establish introduced populations outside their native range (e.g., [29]).

To support further studies related to the worldwide performance of the AI model, we created a Mapping Matrix (MM) describing country-species presence to allow better worldwide regularization, based on The Reptile Database [4].


$${MM}_{cs}=\left\{ \begin{array}{l} 1,\;if\;\mathrm{species}\;S\in \mathrm{country}\;C\\ 0,\;else \end{array}\right.$$ (1)

### The Artificial Intelligence module

The main building block of the AI module is a recent neural network architecture–Vision Transformer (ViT)–with state-of-the-art image categorization performance [30]. Apart from the convolutional neural networks, the ViT avoids convolutional layers while interpreting an image as a sequence of patches and processing it by a standard Transformer encoder like natural language processing.

This section describes the full training and evaluation procedure, including the training strategy and image augmentations. We include the description of used principles that helped to increase the model performance. We include the link to the open-sourced code, trained checkpoints and images, allowing reproducibility for all provided metrics.

### Training strategy

The model was initialized from a publicly available checkpoint (GitHub) and further fine-tuned in two consecutive stages. The PyTorch deep learning framework within the 21.07 NGC Docker container was used. All images were resized to the input size of 224 x 224 or 384 x 384 to match the input resolutions of the pre-trained models.

**Stage1**: Starting from the ImageNet-1k pre-trained checkpoint, we trained the model for 50 epochs. In other words, each image in the training set was feed-forwarded 50 times. For optimization, Stochastic Gradient Descent with momentum set to 0.9 was used. Stochastic Gradient Descent is an optimization algorithm that iteratively modifies the AI model parameters by measuring discrepancies between predicted and correct species names. We used an Adaptive Learning Rate (LR) strategy to schedule the learning rate, i.e., starting LR of 0.01 was reduced by 10% on every second epoch without validation loss reduction. The loss was calculated as Softmax Cross Entropy. To allow better convergence, we accumulated gradients to match a mini-batch size of 256.

**Stage2**: We used both the training and validation set to fine-tune the model in the second stage. In addition, we have substituted the SoftMax Cross Entropy with the Focal Loss that focuses on the hard examples [31]. With that, we prevent the common species with the most samples from overwhelming the model during training. Next, we used the One Cycle Learning Rate Policy, proposed by Smith et al. (2019) [32], to fine-tune the model for an additional 20 epochs.

### Data cleaning

To increase the number of samples for species with few images in online biodiversity platforms, we added weakly labelled data from Flickr (i.e., data with a relatively high number of incorrect species labels). This procedure is commonly used in practice to maximize the number of samples for rare classes and to allow better performance overall as long as the number of incorrect labels does not overwhelm the classifier. To test if the “unreasonable effectiveness” of the weakly labelled data for fine-grained recognition applies to snake recognition as well [21, 22], we trained the ViT-Base/32–224 on the clean (without Flickr data) and full set using the Stage1 training strategy. The experiment results show that including noisy data for rare species improves the performance in all measured metrics (Table 1). Learning from that, we use the Full set to develop the recognition system.

**Table 1:** Performance comparison for two ViT-Base/32 models trained on the SnakeCLEF2021 dataset and its “clean” subset.

	F1 Country	F1—Species	Top1 Accuracy—Species	F1—Genus	Top1 Accuracy—Genus
Clean set	68.6%	69.7%	82.3%	72.5**%**	90.0%
Full set	**75.9%**	**74.2%**	**88.2%**	**77.9%**	**93.4%**

### Data augmentations

**Fig 2:**
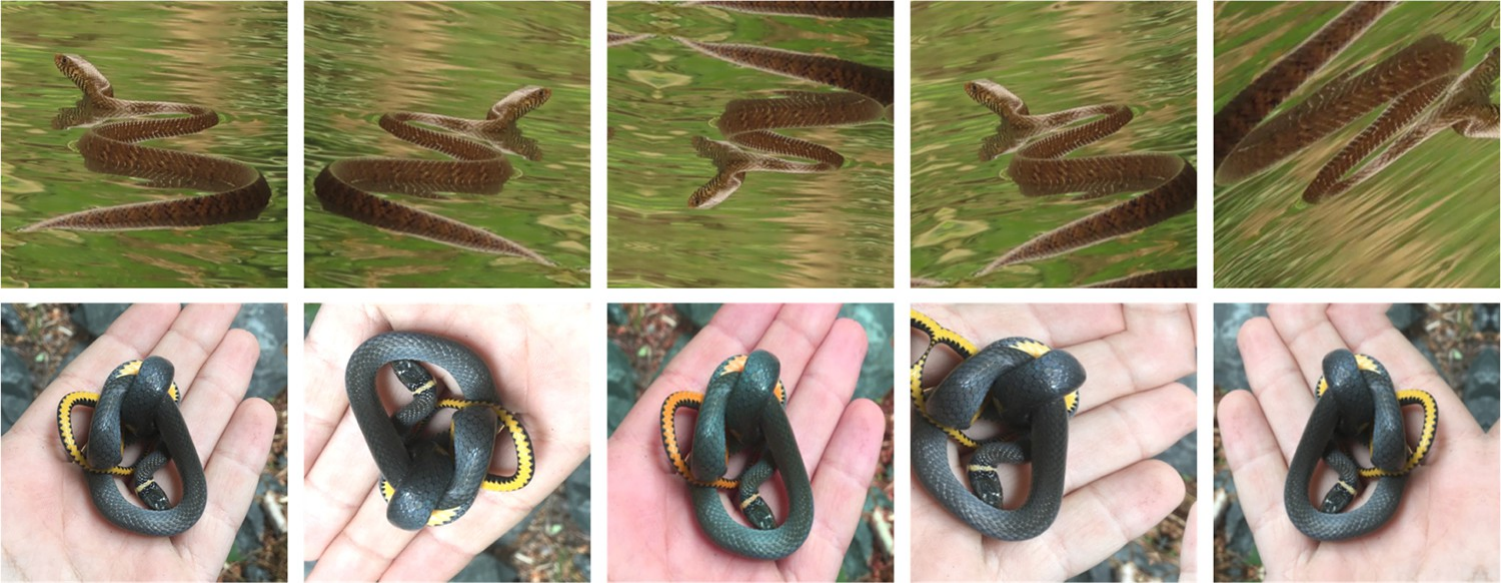
Examples of augmented images. The first image from the left is the original image. Top: image by chiuluan, iNaturalist 207060926 (CC-BY); Bottom: image by Alex Karasoulos, iNaturalist 207359674 (CC-BY).

To prevent overfitting–a state of the model when performing nearly perfect on training data but badly on test data–and to increase the regularization capability of the model, we utilized several augmentation techniques from the Python Albumentations library [33] (Fig 2). During training, we used:

- *RandomResizedCrop*: randomly crops 70–100% from the original image,
- *Horizontal Flip*: flips the image with 50% probability,
- *Vertical Flip*: flips the image with 50% probability,
- *RandomShiftScaleRotate*: shifts, scales and rotates the image with 75% probability, and upper limits of ±6.25%, ±25%, and ±45° respectively,
- *JpegCompression*: changes the image quality with 50% probability on a scale of 50–100,
- *Blur*: using a 7x7 linear filter to blur image with 10% probability,
- *RandomBrightnessContrast*: adjusts the contrast and brightness with 30% probability by a random factor in a range -0.2–0.2,
- *HueSaturationValue*: changes hue, saturation and value of the input image with 20% probability and random limits of -20–20, -30–30, and -20–20%, respectively, and
- *ImageNormalization*: colour values are re-scaled from 0 − 255 to 0 − 1, and normalized by mean (0.5) and std (0.5) in all channels.

### Test-time augmentation

The test-time augmentation (TTA) is a simple procedure commonly used to improve the performance of the neural network. Instead of feeding the model with a single photograph to get the prediction, a batch of augmented images is created and feed-forwarded. In our case, we create a batch of 4 with (i) original image, (ii) horizontally flipped image, (iii) vertically flipped image, and (iv) image rotated by 180°. All four probabilities are averaged to get the prediction.

Such a procedure helps to improve the recognition performance by allowing the algorithm to see the original image on different scales or observed from different angles. The results showing the impact of the TTA are presented in Table 2.

**Table 2:** Test time augmentations experiment.

	F1 Country	F1—Species	Top1 Accuracy—Species	F1—Genus	Top1 Accuracy—Genus
Baseline	91.1%	88.8%	94.1%	**93.2%**	98.4%
Test-time augmentation	**91.3%**	**89.1%**	**95.2%**	92.5%	**98.5%**

### Exploiting geographic information

We tested and utilised two approaches for the integration of geographic information—binary masking, which automatically removes all the irrelevant species for a country from the prediction and a simple statistical approach based on the assumption that the class posterior given the image I and location L can be estimated as:

$$P(S|I,\;L)=\frac{P(S|I)∙P(S|L)}{P(S)},$$ (2)

where p(S) is the species prior in the training set, and the conditional probability P(S|L) is calculated as the relative frequency of species S within the given location.

The first scenario, where we removed all the irrelevant SoftMax values based on the species-presence knowledge, helped us to increase the performance by a significant margin; 2.93% and 3.11% in F1 Country and F1—Species, respectively (Table 3).

**Table 3:** Achieved performance on the SnakeCLEF2021 test set using different locational data and metadata integration method.

	F1 Country	F1—Species	Top1 Accuracy—Species	F1—Genus	Top1 Accuracy—Genus
Model with Test-time augmentation	91.3%	89.1%	95.2%	92.5%	98.5%
Country Prior	90.0%	89.1%	95.4%	91.5%	98.6%
Continent Prior	93.2%	90.7%	95.1%	**95.6%**	98.9%
Presence Masking	**94.2%**	**92.2%**	**96.0%**	94.9%	**99.0%**

### Evaluation Protocol

To assure focus on worldwide performance, we defined the macro F1 country performance (Macro F1_C_) as the main metric. We calculate it as the mean of country F1 scores:

$${Macro\;F1}_{c}=\frac{1}{N}\sum_{c=1}^{N}\;{F1}_{c},\;{F1}_{c}=\frac{1}{\sum_{s=1}^{N}\;{\mathrm{MM}}_{cs}}\sum_{s=1}^{N}{F1}_{s}∙{\mathrm{MM}}_{cs}$$ (3)

where C is country index, S is species index, F1_C_ is the country performance, and MM_CS_ is the mapping matrix describing species-country presence that allows better worldwide regularization; extracted from the August 2020 release of The Reptile Database [4].

To get the F1_S_ we use the following formula for each species:

$${F1}_{s}=2∙\frac{P_{s}\times R_{s}}{P_{s}+R_{s}},\;P_{s}=\frac{{tp}_{s}}{{tp}_{s}+{fp}_{s}},\;R_{s}=\frac{{tp}_{s}}{{tp}_{s}+{fn}_{s}}$$ (4)

To allow deeper comparison on different levels, we also measure the Top1 Accuracy ($\frac{\#Correct\;Assessments}{\#All\;Assessments}$) and the Macro F1 score. The Macro F1 score is calculated as the mean of all F1_S_ scores:

$$Macro\;F1=\frac{1}{N}\sum_{s=1}^{N}{F1}_{s},$$ (5)

where *S* is the species index and *N* the number of species. Final Macro F1 is calculated by computing the F1 score for each species as the harmonic mean of the species Precision (*P*_*S*_) and the Recall (*R*_*S*_).

## Results

### Model overall performance

We tested the performance of the model on an independent dataset comprising 23,673 photos. The model is based on the novel neural network architecture–Vision Transformer–and was optimized using various data augmentations (i.e., random crop, horizontal/vertical flip, random rotation, etc.). In addition, the test-time augmentation procedure is used in the production environment. We also incorporated geographic metadata information, which increased the system’s performance by a significant margin, reducing the relative error rate by 33.3%.

The model accurately classifies testing images at the species and genus levels (Table 4). The model macro-averaged F1 score, calculated as the mean of all species F1 scores, is 92.2% and the top-1 accuracy is 96.0%. For genus recognition, the model achieves a macro-averaged F1 score of 94.9% and a 99.0% top-1 accuracy. The macro F1 country performance, calculated as the mean of country F1 scores, is 94.2%.

**Table 4:** Overall performance of the model.

Test Set	F1 Country	F1—Species	Top1 Accuracy—Species	F1—Genus	Top1 Accuracy—Genus
SnakeCLEF2021	94.2%	92.2%	96.0%	94.9%	99.0%

### Relationship between number of training images per species and F1 score

**Fig 3:**
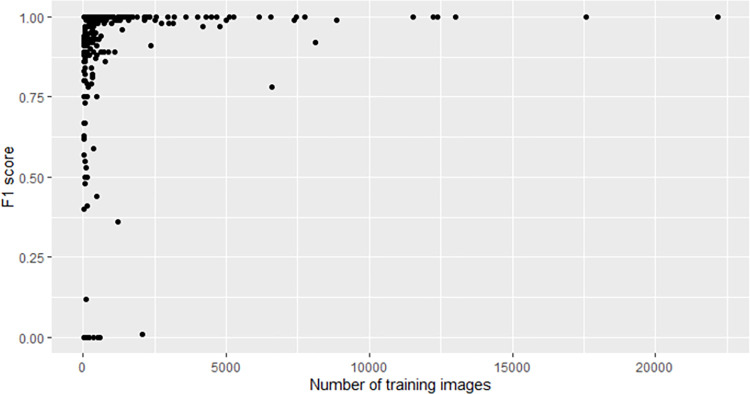
Distribution of F1 scores according to the number of training images per species. See S2 Data for underlying values.

There is a logarithmic relationship between the number of training images per species and the F1 score (Fig 3). Species that stand out as being relatively inaccurately identified for their quantity of training data are mostly those which have been recently delineated primarily using molecular methods and geographic location rather than morphological characteristics, and previously belonged to more widespread species complexes, such as *Agkistrodon piscivorus/A*. *conanti*, *Boa constrictor/B*. *imperator*, and *Salvadora hexalepis*/*S*. *deserticola*. Durso et al. (2021) [34] found the same and noted that training images for these species complexes are much more likely to be mislabeled due to confusion over how best to differentiate the putative species, especially from photos that lack geographic locality information. From the probabilistic point of view, the species with fewer training images will normally be mistaken for those with more.

Species having good accuracy despite relatively low quantity of training data include diverse species from different clades and continents. For instance, Bandy-bandy (*Vermicella annulata*), an Australian burrowing elapid with bold black-and-white bands, had an F1 = 1 and only 522 training images. Although there are other banded snakes in Australia [35], *V*. *annulata* is among the most distinctive Australian snake species and is also notable for its body-bridging defensive behaviour [36], which might provide unique features (e.g. particular postures) that the algorithm could detect. However, there are five other species in the genus *Vermicella*, all of which are similar in appearance but none of which met the minimum threshold of ten training images, and we suspect that including these would produce at least occasional confusion among them, lowering the F1 for *V*. *annulata*. The same applies to many other diverse genera represented in our training data by only one species that met the 10-image threshold.

### Country-level performance of the model

**Fig 4:**
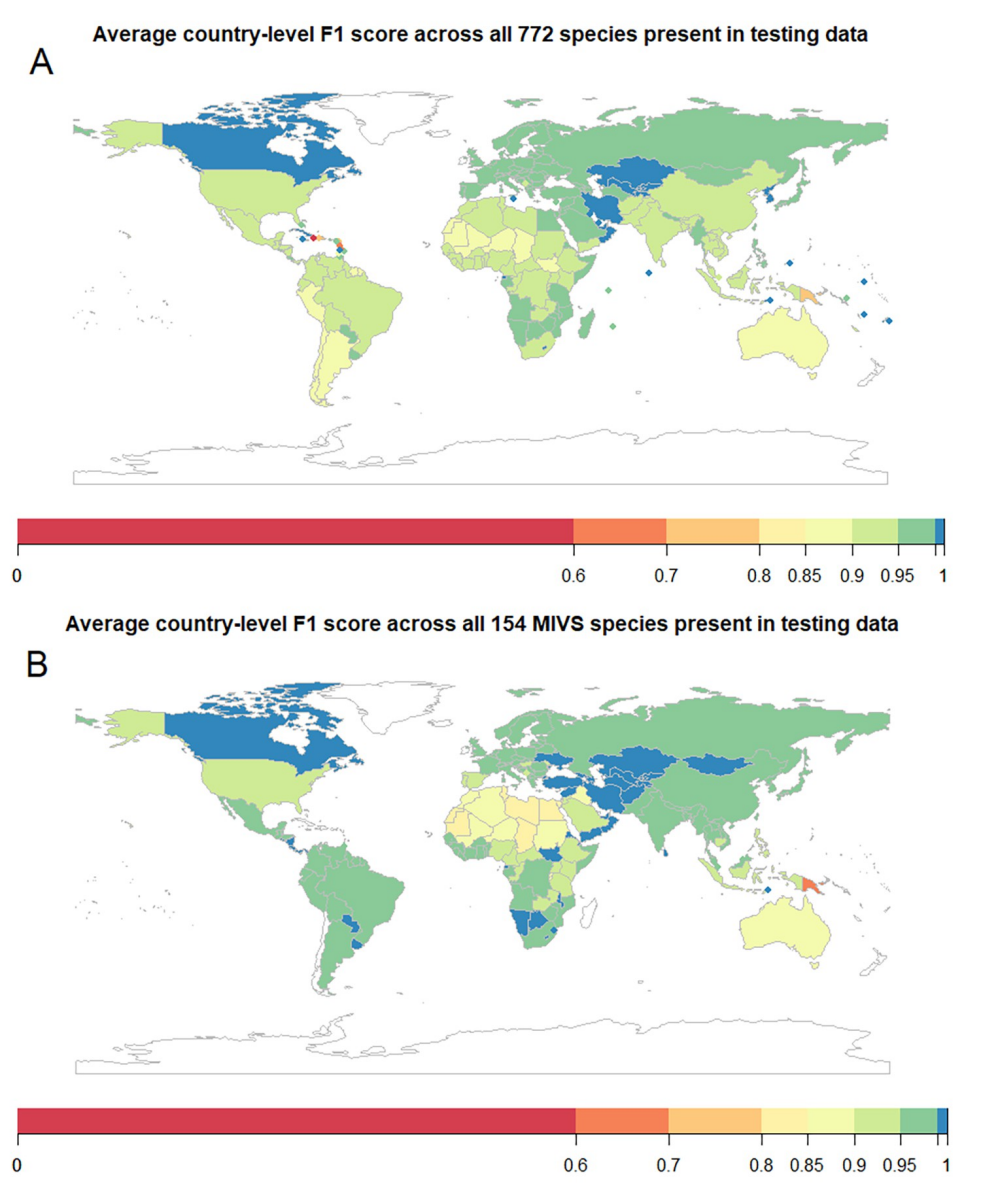
Maps of average F1 score by country (A All species, B MIVS only). Countries and territories with the lowest overall F1 scores for all species are Martinique (36%), Haiti (40%), Dominica (62.5%), St. Lucia (62.5%), Aruba (68%), and Papua New Guinea (74.8%). The map was drawn using R package rworldmap v 1.3.6 [26]. See S3 and S4 Data for panels A and B underlying values.

We assessed the country-level performance of the model, taking into account the list of snake species occurring in each country. The model’s country-wise F1 is above 70% in 97% of countries, above 90% in 88% of countries, and above 95% in 56% of countries (Fig 4).

**Fig 5:**
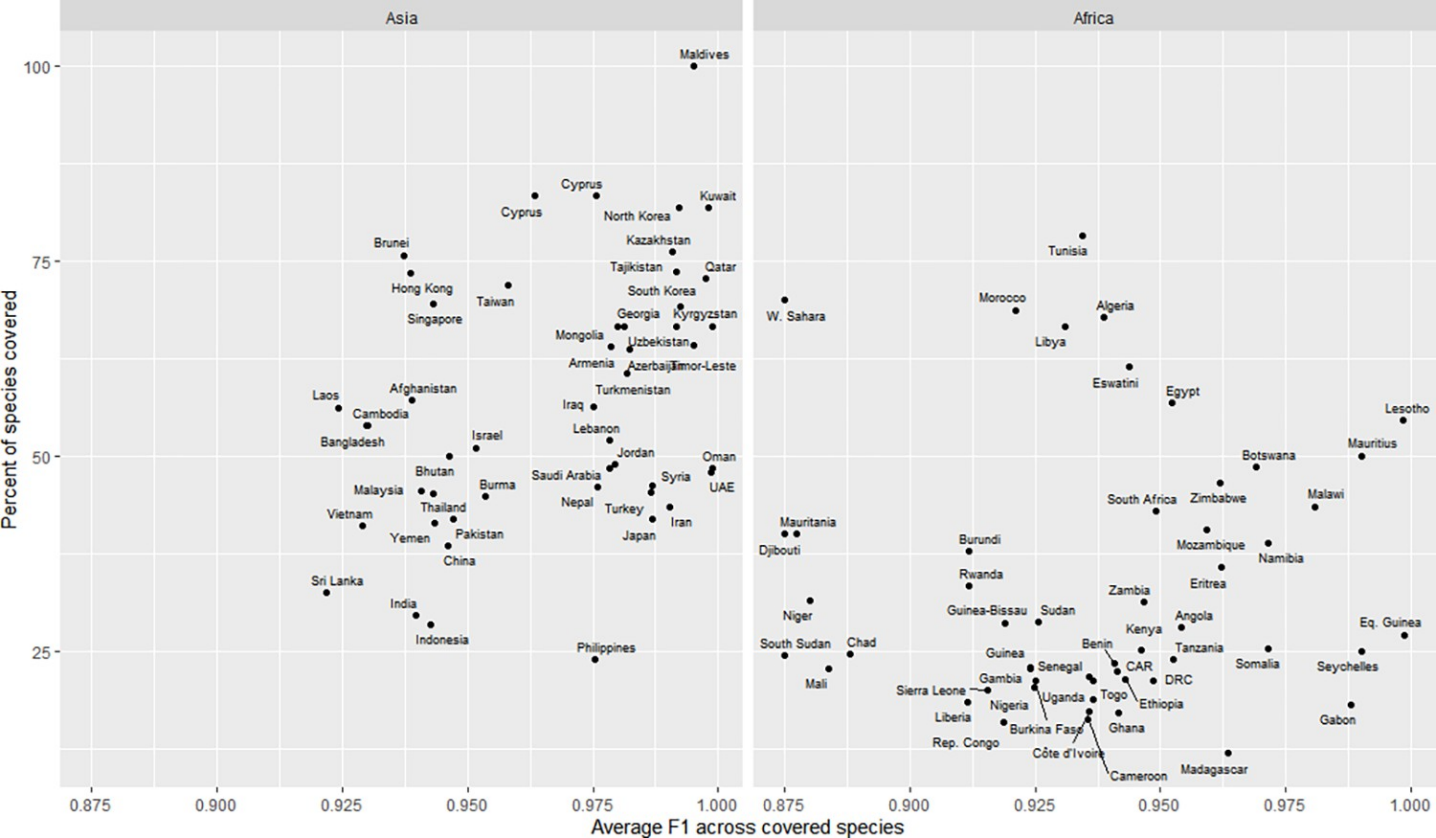
Scatterplot of Asian and African countries by coverage and F1 score. See S5 Data for underlying values. For other continents, see S3 Fig. and interactive online version at https://chart-studio.plotly.com/~amdurso/6/#/.

There is a trade-off between the model coverage and performance that varies by region. Focusing on the two continents with the highest snakebite burden, we found that, in Asia, countries with higher coverage have higher performance on average, whereas in Africa, performance peaks at intermediate coverage (Fig 5).

### Comparing model performance between MIVS and non-MIVS species

**Fig 6:**
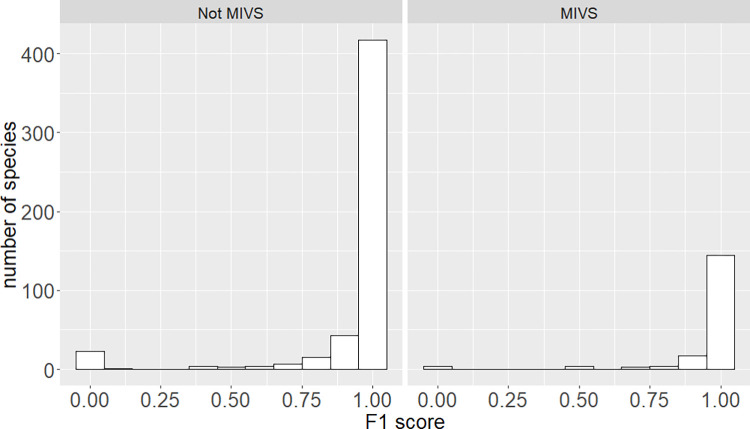
Comparison of F1 score distribution between non-MIVS (574 species) and MIVS (198 species). See S6 Data for underlying values.

The 772 species used to train the model include 198 MIVS and 574 non-MIVS species. We compared the model performance in identifying MIVS and non-MIVS species (Fig 6). The model performed equally at identifying MIVS and non-MIVS, with a similar F1 score distribution for each category (average ± S.D. F1 = 95 ± 17% for MIVS *vs* 93 ± 22% for non-MIVS; p = 0.61).

### Model performance in distinguishing snake lookalikes

**Fig 7:**
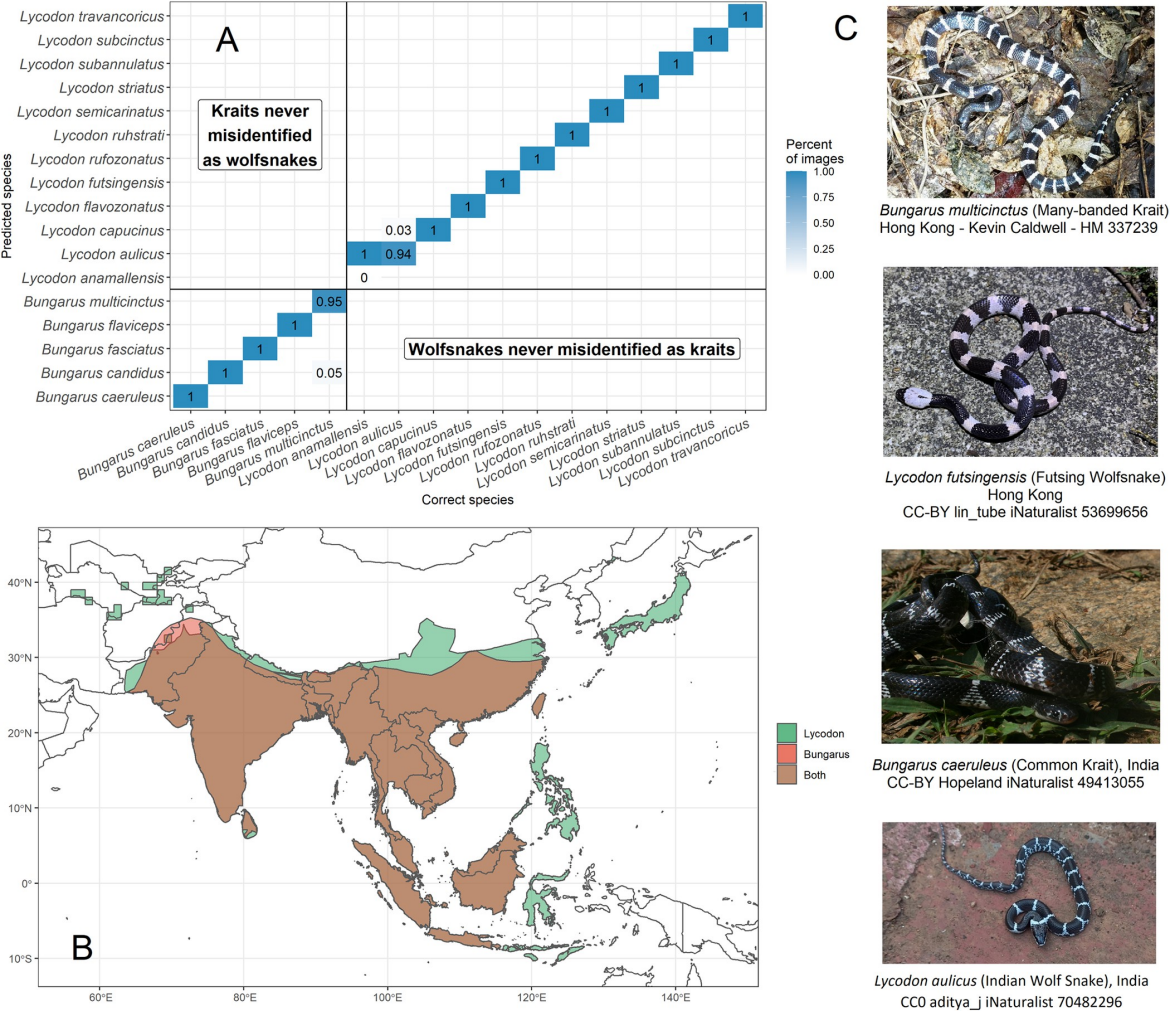
Computer vision model performance for identifying lookalike venomous and non-venomous snake species in Southeast Asia (*Bungarus spp*. vs. *Lycodon spp*). All species in these genera from the training data were included. **A** Confusion matrix of the classification results of the model on lookalike snake species with the percentage (blue colour code) of correctly (diagonal cells) and incorrectly (off-diagonal cells) classified images, **B** Geographic range of the relevant snake genera based on [25], **C** Representative photos of some of the lookalike snake species tested with the model. The map was drawn using R package rworldmap v 1.3.6 [26]. See S7 Data for panel A underlying values.

**Fig 8:**
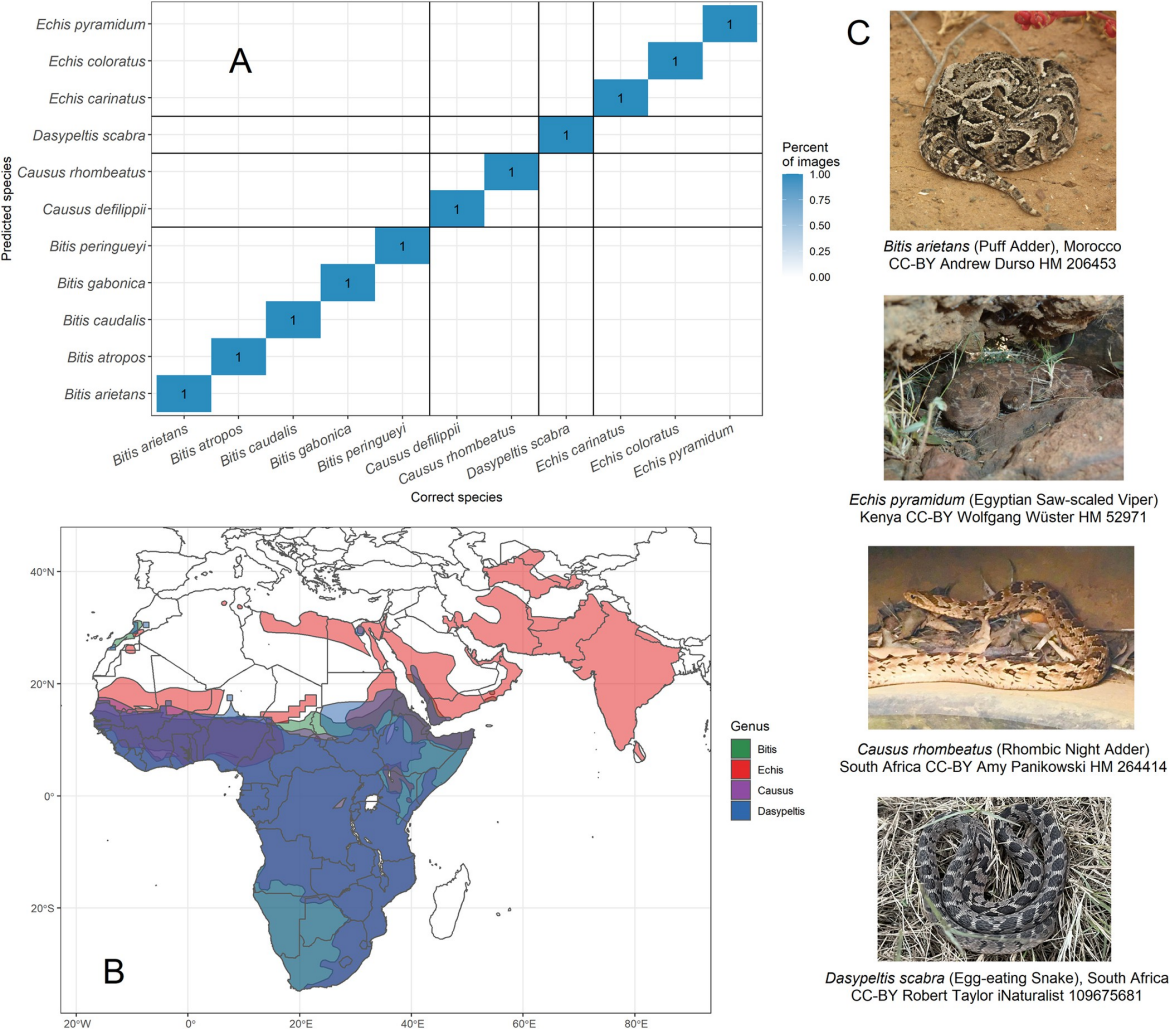
Computer vision model performance for identifying lookalike venomous and non-venomous snake species in Sub-Saharan Africa (*Bitis spp*. vs. *Echis spp*. vs. *Causus spp*. vs. *Dasypeltis spp*.). All species in these genera from the training data were included. **A** Confusion matrix of the classification results of the model on lookalike snake species with the percentage (blue colour code) of correctly (diagonal cells) and incorrectly (off-diagonal cells) classified images, **B** Geographic range of the relevant snake genera based on [25], **C** Representative photos of some of the lookalike snake species tested with the model. The map was drawn using R package rworldmap v 1.3.6 [26]. See S8 Data for panel A underlying values.

The training dataset includes many similar-looking species, including several non-MIVS species that have evolved to mimic MIVS species. These species usually occur in the same geographic area as each other and resemble one another so closely that they are often confused, even by experts [9, 37]. We assessed the model performance in distinguishing selected lookalike species groups occurring in Southeast Asia (Fig 7) and sub-Saharan Africa (Fig 8).

In Southeast Asia, one of the most difficult medically-relevant snake identification challenges is telling apart venomous kraits (genus *Bungarus*) from non-venomous wolfsnakes (genus *Lycodon*). Confusion between these two groups of snakes has led to unnecessary or delayed use of antivenom, resulting in victim death [5, 9, 38]. Whereas >91% of community members in southern Nepal confused these two groups [39], our model accurately classified all testing photos at the genus level (although it did confuse some species within each genus). Several species in each genus remain unrepresented in the training data.

In Sub-Saharan Africa, similar-looking venomous genera (*Bitis* and *Echis*) and mildly venomous (*Causus*) or non-venomous (*Dasypeltis*) lookalikes are frequently confused [37]. Our model accurately classified all testing images at the species level. However, few testing photos were available for many taxa, and several species in each genus remain unrepresented in the training data.

## Discussion

This study shows for the first time that AI (i.e., computer vision and fine-grained image classification) can identify a large diversity of venomous and non-venomous snakes at a high level of accuracy. The taxonomic and geographic coverage and the performance of the computer vision model proposed are unprecedented: 772 snake species and 269 genera from 188 countries on six continents and an F1-macro score of 92.2%, with a species and genus level accuracy of 96.0% and 99.0%, respectively. This study sets the foundation for developing global, regional or national snake identification systems for herpetologists, epidemiologists and healthcare providers, and the general public, and highlights the importance of AI, open data, and crowdsourcing to tackling snakebite.

For developing this new AI model, we used snake photos from nearly every country (188 out of 215). This global approach contrasts with previous studies focused on a specific country (India, Indonesia, Iran, Malaysia, or Sri Lanka) (e.g., [16, 18]), a geographic area (Galapagos Islands) [17], or a limited number of species (e.g., 6 in Rajabizadeh et al. (2021) [18], 9 in Patel et al. (2020) [17], 22 in Amir et al. (2016) [16]). AI-based snake photo classification is challenging, mainly because of the large diversity of snakes and the often-limited number of photos per class. Thus collaborative approaches and open data sharing, like in biodiversity platforms such as HerpMapper and iNaturalist, are essential. These platforms also provide promising AI models for classifying snakes, although they do not deliver snake-specific performance metrics.

We developed part of our model collaborating with an international community of AI scientists via AICrowd and the SnakeCLEF2021 challenge [40], and building on solutions for classifying fungi [41]. Our model is based on Vision Transformer, the state-of-the-art deep neural network, and uses simple and replicable training procedure and unique geographic data exploitation. With an F1 Country of 94.2%, F1 species of 92.2%, and Top1 accuracy of 96.0%, there is, to our knowledge, no other AI-based system offering similar performance, even for a smaller number of snake species.

The average model accuracy per country is above 90% in 88% of countries. Accurate taxonomic identification of snakes is critical to improving snake ecology and snakebite epidemiology data in endemic countries. Understanding the diversity of snakes and which snakes bite locals is the basis for more precise and cost-effective distribution of often-limited national stock of antivenoms. This is also the rationale for WHO’s “Snakebite Information and Data Platform” and the associated sub-Saharan Africa antivenom stockpile programme [24].

Snakebite is a health emergency requiring rapid transfer of the victim to a healthcare facility (e.g., neurotoxic envenomation can produce generalized paralysis, respiratory arrest, and death in few hours). Precise and rapid snake identification could help healthcare providers anticipate victim signs and symptoms, complement the commonly used syndromic approach, make good choices on the use of antivenom or plan victim transfers depending on antivenom availability. Accurate snake species identification is particularly important in snakebite endemic countries and regions where species-specific monovalent antivenoms are used to treat victims. Our AI model distinguishes lookalike venomous and non-venomous species often confused in clinical practice with potentially severe implications for the victim (e.g., wolfsnakes (*Lycodon spp*.) and kraits (*Bungarus spp*.)) [5, 9, 38]. Existing snake identification techniques (e.g. immunoassays, PCR-based tests) require laboratory capacity and lengthy and costly procedures and are thus not yet adapted to low-resource snakebite endemic countries [10]. In certain countries, healthcare providers rely on local, national and even international herpetologists for identifying snakes brought to the health facility or photographed by the victims or relatives, as recommended by WHO (e.g., [14]). Although some specific platforms provide snake identification support (e.g., Sri Lanka (www.snakesidentification.org) [42], Thailand (www.thailandsnakes.com/thailand-snake-id/) [24]), an informal and unsecured process has emerged among healthcare providers, who share snake photos with other medics or herpetologists for identification via email, WhatsApp or even Facebook. Herpetologists usually provide their expertise on a volunteer basis and their availability and reactivity may be limited. In this context, AI could deliver simple, continuous, instantaneous, and low-cost snake identification to complement herpetologists [37, 43] and support healthcare providers and snakebite victims, especially considering the rapidly growing internet and mobile technology penetration in many snakebite endemic countries [44] (e.g., India). Improving the capacity of communities at risk of snakebite, conservationists, nature lovers, among others groups, to identify and learn about snakes could both help prevent snakebite envenoming (e.g., by recognising venomous snakes) and protect snakes (e.g., fewer snakes will be killed out of fear or ignorance).

Other professional groups, such as snake rescuers, veterinarian practitioners confronted to snakebite in companion and livestock animals [45], and wildlife trade inspectors (e.g., 6.3 million snakes were traded globally between 1975 and 2018 [27]), or biology students, snake enthusiasts and travellers, could also benefit from this AI.

Our study has several limitations. First, the snake photo dataset used to train the model does not yet cover the whole species diversity of some highly biodiverse regions: the mean percentage of species covered per country in Asia is 57%, 44% for Oceania, 41% for Latin America/Caribbean, and 34% for Africa. We have shown that online communities of snake enthusiasts and herpetologists can contribute large volumes of geo-tagged snake observations [19] and that aggregating photos among online data sources brought our team to the leading edge of global snake image datasets, with almost 80% of the world’s species represented by at least one image [20]. Yet, only 20% of the world’s species met our 10-image threshold for inclusion in the training dataset, highlighting the extremely long-tailed distribution of photos per species. Although online data sets continue to grow, connecting with difficult-to-access online (e.g. sub-communities of Facebook, Field Herp Forum) and offline (e.g. private WhatsApp snake identification chat groups, private or natural history museum image collections) communities will allow us to progress in snake identification. The species under-represented in our snake photo dataset are shown in S1 Fig and S3 Table and the most-wanted species globally in [20]. To help improve the model accuracy and coverage, we encourage professional herpetologists and snake enthusiasts with photos of these species or missing species to submit them to The Reptile Database [4] or the citizen science biodiversity platforms iNaturalist and HerpMapper. Second, the snake photos used to evaluate the model performance (iNaturalist photos) may be "easy" for the algorithm to correctly identify and not reflect the kinds of photos that are usually taken in the context of a snakebite event (e.g., the biting snake has been killed and its head smashed, the photo is blurred, only part of the snake’s body is visible, etc.). Further research is needed to test this AI snake identification system in the field. Third, we compared a limited number of lookalike species that we selected based on our literature review [9] and discussions with clinicians (GA). Further studies need to include and more systematically compare lookalike snake species occurring at the global scale.

## Conclusion

We have built and openly shared an international AI model for the automatic identification of snakes, setting the basis for further machine learning research and AI applications to tackling snakebite and other neglected tropical diseases in low-resource settings. The power of AI must be embraced and used safely and equitably to improve health and wellbeing of the poorest communities of the world.

## Supporting information

S1 Table SnakeCLEF 2021 training set. Data sources and their taxonomic and geographic coverage.
(DOCX)

S2 Table Details of the datasets used for training and testing of the model. (DOCX)

S3 Table MIVS species included in the training and test datasets. (XLSX)

S1 Fig Number of training photos per MIVS species. (PDF)

S2 Fig Number of test photos per MIVS species. (PDF)

S3 Fig Scatterplot of countries by coverage and accuracy. See S9 Data for underlying values. See interactive online version at https://chart-studio.plotly.com/~amdurso/6/#/.
(TIFF)

S1 Data CSV file containing the underlying numerical data for Fig 1. (CSV)

S2 Data CSV file containing the underlying numerical data for Fig 3. (CSV)

S3 Data CSV file containing the underlying numerical data for Fig 4A. (CSV)

S4 Data CSV file containing the underlying numerical data for Fig 4B. (CSV)

S5 Data CSV file containing the underlying numerical data for Fig 5. (CSV)

S6 Data CSV file containing the underlying numerical data for Fig 6. (CSV)

S7 Data CSV file containing the underlying numerical data for Fig 7. (CSV)

S8 Data CSV file containing the underlying numerical data for Fig 8. (CSV)

S9 Data CSV file containing the underlying numerical data for S3 Fig. (CSV)
