# Supplementary material for: An artificial intelligence model to identify snakes from across the world: Opportunities and challenges for global health and herpetology
Source: PLoS Negl Trop Dis. 2022 Aug 15;16(8):e0010647. doi: 10.1371/journal.pntd.0010647 (PMC9426939; doi:10.1371/journal.pntd.0010647)
Supplement: S1 Table — Data sources and their taxonomic and geographic coverage. (DOCX) [file pntd.0010647.s001.docx]

**S1 Table. SnakeCLEF 2021 training set - Data sources and their taxonomic and geographic coverage**

|  | # of Species | # of Genera | # of Families | # of photos | # of Countries |
| --- | --- | --- | --- | --- | --- |
| iNaturalist | 762 | 265 | 17 | 277,025 | 181 |
| HerpMapper | 614 | 244 | 17 | 58,351 | 98 |
| Flickr | 733 | 260 | 18 | 50,630 | 125 |
| **All** | **772** | **269** | **18** | **386,006** | **188** |
