# Supplementary material for: An artificial intelligence model to identify snakes from across the world: Opportunities and challenges for global health and herpetology
Source: PLoS Negl Trop Dis. 2022 Aug 15;16(8):e0010647. doi: 10.1371/journal.pntd.0010647 (PMC9426939; doi:10.1371/journal.pntd.0010647)
Supplement: S2 Table — (DOCX) [file pntd.0010647.s002.docx]

**S2 Table. Details of the datasets used for training and testing of the model**

|  | # of Species | # of Genera | # of Families | # of photos | # of Countries |
| --- | --- | --- | --- | --- | --- |
| Training Set | 772 | 269 | 18 | 386,006 | 188 |
| SnakeCLEF2021 Test Set | 694 | 249 | 17 | 23,673 | 134 |
