# Supplementary figures and images for: An artificial intelligence model to identify snakes from across the world: Opportunities and challenges for global health and herpetology
Source: PLoS Negl Trop Dis. 2022 Aug 15;16(8):e0010647. doi: 10.1371/journal.pntd.0010647 (PMC9426939; doi:10.1371/journal.pntd.0010647)

Species Label frequency

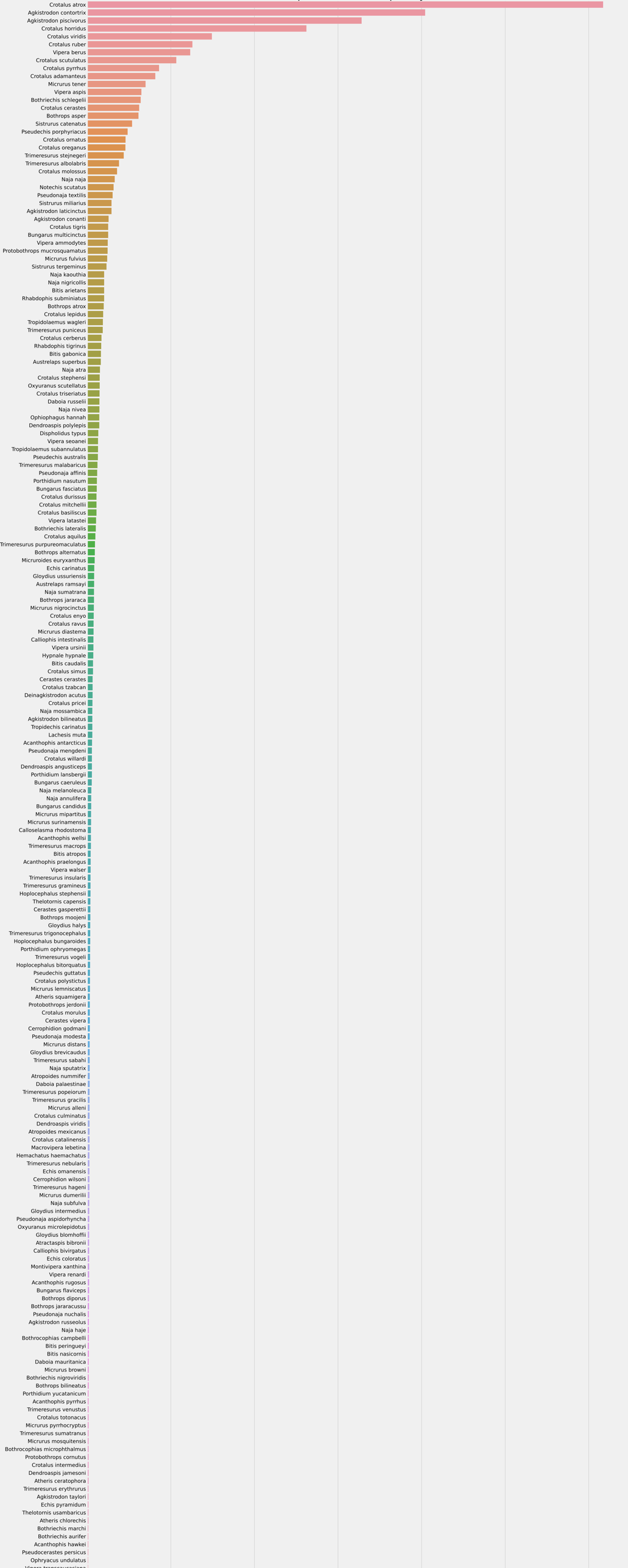

Supplement: S1 Fig — (PDF) [file pntd.0010647.s004.pdf]

Species Label frequency

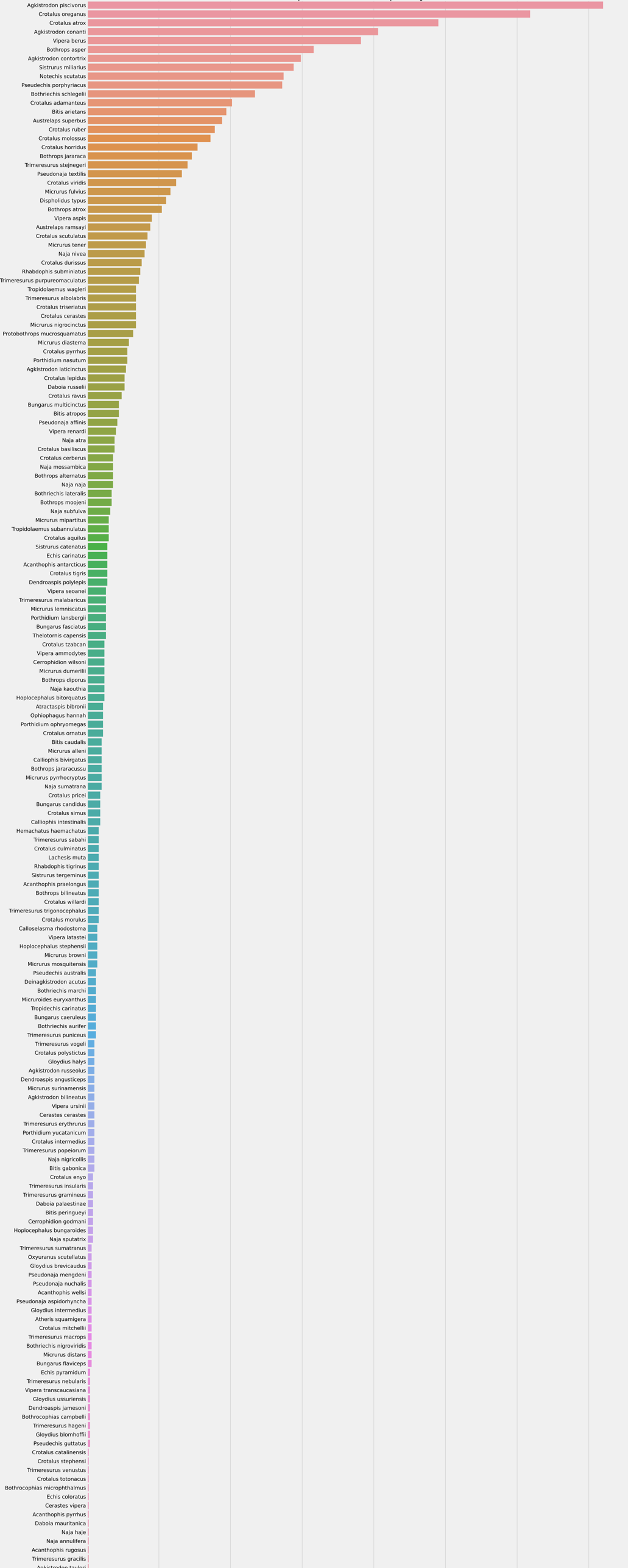

Supplement: S2 Fig — (PDF) [file pntd.0010647.s005.pdf]

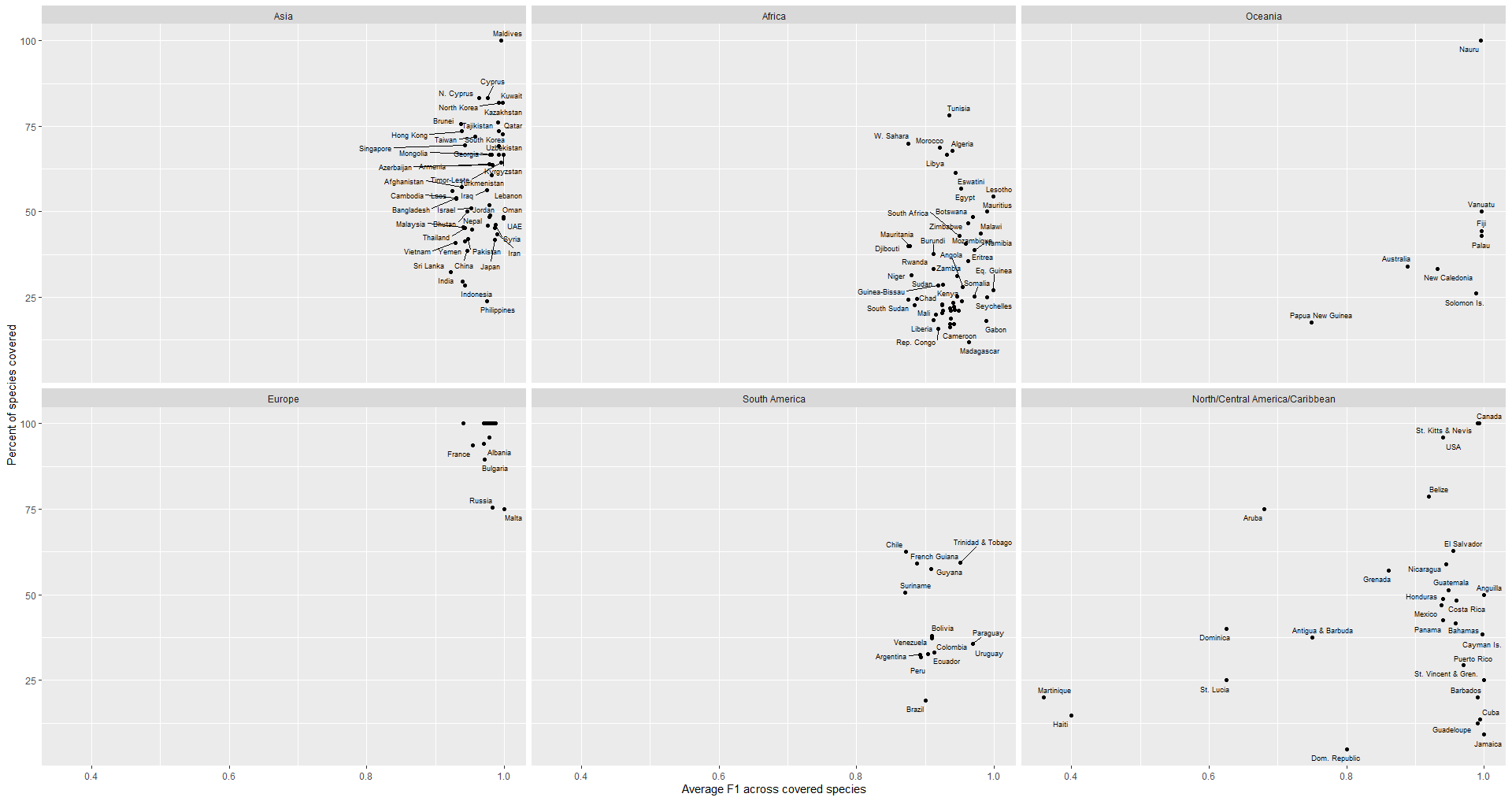

Supplement: S3 Fig — See S9 Data for underlying values. See interactive online version at https://chart-studio.plotly.com/~amdurso/6/#/. (TIFF) [file pntd.0010647.s006.tiff]
